# Evaluation of short interval cortical inhibition and intracortical facilitation from the dorsolateral prefrontal cortex in patients with schizophrenia

**DOI:** 10.1038/s41598-017-17052-3

**Published:** 2017-12-06

**Authors:** Yoshihiro Noda, Mera S. Barr, Reza Zomorrodi, Robin F. H. Cash, Faranak Farzan, Tarek K. Rajji, Robert Chen, Zafiris J. Daskalakis, Daniel M. Blumberger

**Affiliations:** 1 Temerty Centre for Therapeutic Brain Intervention, Centre for Addiction and Mental Health, Toronto, Ontario, M6J 1H4 Canada; 2 Department of Psychiatry, University of Toronto, Toronto, Ontario, M5T 1R8 Canada; 3 Campbell Family Mental Health Research Institute, Centre for Addiction and Mental Health, Toronto, Ontario, M5T 1R8 Canada; 4 Division of Neurology, Department of Medicine, University of Toronto, Division of Brain, Imaging and Behaviour – Systems Neuroscience, Krembil Research Institute, University Health Network, Toronto, M5T 2S8 Ontario, Canada

## Abstract

GABAergic and glutamatergic dysfunction in the dorsolateral prefrontal cortex (DLPFC) are thought to be the core pathophysiological mechanisms of schizophrenia. Recently, we have established a method to index these functions from the DLPFC using the paired transcranial magnetic stimulation (TMS) paradigms of short interval intracortical inhibition (SICI) and facilitation (ICF) combined with electroencephalography (EEG). In this study, we aimed to evaluate neurophysiological indicators related to GABA_A_ and glutamate receptor-mediated functions respectively from the DLPFC in patients with schizophrenia using these paradigms, compared to healthy controls. Given that these activities contribute to cognitive functions, the relationship between the TMS-evoked potential (TEP) modulations by SICI/ICF and cognitive/clinical measures were explored. Compared to controls, patients showed reduced inhibition in P60 (t_22_ = −4.961, p < 0.0001) by SICI and reduced facilitation in P60 (t_22_ = 5.174, p < 0.0001) and N100 (t_22_ = 3.273, p = 0.003) by ICF. In patients, the modulation of P60 by SICI was correlated with the longest span of the Letter-Number Span Test (r = −0.775, p = 0.003), while the modulation of N100 by ICF was correlated with the total score of the Positive and Negative. Syndrome Scale (r = 0.817, p = 0.002). These findings may represent the pathophysiology, which may be associated with prefrontal GABA_A_ and glutamatergic dysfunctions, in the expression of symptoms of schizophrenia.

## Introduction

Schizophrenia is a chronic and devastating mental disorder, which is a leading cause of years of life lost to disability^1^. Antipsychotics usually alleviate positive symptoms of patients with schizophrenia, however, the effects on the negative symptoms or cognitive impairments are limited^2^. The pathological mechanisms underlying these symptoms in schizophrenia are not fully elucidated, though, impairments in gamma-aminobutyric acid (GABA) receptor-mediated inhibitory and glutamate receptor-mediated excitatory neurotransmissions are strongly implicated^3^. Reduced GABA receptor-mediated inhibitory neurotransmission is a consistent finding in schizophrenia^4,5^, while decreased glutamate levels in the medial frontal region and glutamate receptor dysfunction are also shown in chronic schizophrenia^6^.

Paired pulse transcranial magnetic stimulation (TMS) is a non-invasive tool that can index neurophysiological property associated with neurotransmissions in the cortex^7^. Short interval intracortical inhibition (SICI) is one of the TMS neurophysiological paradigms^8^. The neurobiological basis of SICI has been identified through pharmacological studies combined with TMS. For example, it has been shown that GABA_A_ agonist enhance SICI^9,10^, whereas GABA reuptake inhibitor tiagabine decreases SICI^11^. Thus, it is thought that SICI can index GABA receptor-mediated inhibitory function in the motor cortex^7^. In contrast, intracortical facilitation (ICF) is one of the facilitatory TMS neurophysiological paradigm^8,12^. Specifically, pharmacological studies with TMS have been demonstrated that NMDA antagonist decreases ICF^13,14^, while GABA_A_ agonist also decreases ICF^10,15^. Thus, ICF is thought to be mainly associated with glutamate receptor-mediated excitatory functions in the motor cortex. Direct evidence of neurotransmitter functioning from combined TMS-EEG from the DLPFC remains relatively unexplored.

In patients with schizophrenia, a reduction of SICI in motor cortex, which is thought to be associated with GABA_A_ receptor-mediated inhibition, has been a replicated finding^5^. Further, such deficits of the motor cortex in schizophrenia may be attributed to global disturbances in GABA receptor-mediated inhibitory neurotransmission, supported by findings of substantial reductions in GABA receptor-mediated inhibitory interneurons, GABA-synthesizing enzyme glutamic acid decarboxylase (GAD67), and GABA-related gene expression in post-mortem studies^3^. In contrast, intracortical facilitation (ICF) in motor cortex appears to be unaltered in patients with schizophrenia^16,17^, despite genetic and pharmacological studies suggesting potential deficits of glutamate-mediated excitatory neurotransmission through the N-methyl-D-aspartic acid (NMDA) receptor in schizophrenia^18^. Furthermore, postmortem studies have demonstrated decreased transcription levels and enzyme activity for glutamate carboxy peptidase in the dorsolateral prefrontal cortex (DLPFC) in patients with schizophrenia, which in turn results in reduced NMDA receptor-mediated signaling^19^. Taken together, dysfunction of GABA receptor-mediated inhibitory as well as glutamate receptor-mediated excitatory neurotransmissions in schizophrenia may underlie an impaired excitatory and inhibitory (E/I) balance in this disorder^20^.

Cognitive impairment is a core symptom of schizophrenia^21^ that is strongly associated with DLPFC dysfunction^22,23^. For example, DLPFC is involved in working memory^24^ and has been targeted to improve working memory deficits^25^ in addition to negative symptoms in schizophrenia^26^. We recently reported that greater working memory performance was associated with greater GABA_A_ receptor-mediated activity indexed by SICI in motor cortex^25^. Recent technological advances allow us to investigate cortical integrity in a non-invasive way utilizing concurrent TMS-EEG^27–29^. Further, it can be possible to more directly investigate the relationship between working memory performance and neurophysiological property associated with GABA_A_ receptor-mediated function in the DLPFC described below, which has not been determined yet.

Given that SICI and ICF deficits may represent underlying neurobiological dysfunction in schizophrenia, evaluating these TMS neurophysiology from the DLPFC and their associations with cognition may further understand the pathophysiology underlying schizophrenia. We have developed a method to measure the strength of inhibitory and excitatory neurotransmissions as indexed by SICI and ICF in the DLPFC using combined TMS–EEG^30^. This technique also allows us to determine the effect of SICI and ICF on individual components and frequency band activities of the TMS-evoked potential (TEP) among patients with schizophrenia compared to healthy controls. In the present study, we hypothesized that: (1) inhibitory and excitatory functions in the DLPFC as measured by the paired pulse TMS paradigms of SICI and ICF would be reduced in patients with chronic schizophrenia compared to the healthy controls; (2) in patients group, reduced SICI and ICF would correlate with cognitive and clinical measures, specifically working memory performance and clinical symptom severity based on previous research^31,32^. The aims of this study were to measure SICI and ICF from the DLPFC in patients with schizophrenia compared to controls and to determine if TEPs induced by these paradigms are associated with working memory performance and clinical symptom severity. In addition, we aimed to explore the modulatory effects on frequency bands by SICI and ICF between patients and controls.

## Results

### Demographic, clinical and cognitive measures, and medication information

Demographic of the patients and controls, and clinical and cognitive measures of the patients are detailed in Table 1. Of note, there was no correlation between the chlorpromazine equivalent dose that each patient was taking during the study and the clinical or cognitive measures. The psychotropic medications of the patients are shown in Supplementary Table.

**Table 1:** Demographic, clinical, and cognitive data of the patients and controls.

Mean (±SD)	Patients (N = 12)	Controls (N = 12)
Age	41 ± 10	39 ± 12
Male: Female	8: 4	6: 6
Years of Education	15 ± 3	15 ± 2
PANSS		
Positive	11.3 ± 3.0	—
Negative	11.7 ± 3.4	—
General	23.6 ± 2.8	—
Total	50.1 ± 6.2	—
WTAR FS IQ	106 ± 10	—
Letter-Number-Span	12 ± 3	—
HVLT		—
Retention (%)	83.4 ± 14.8	—
Discrimination Index	10.5 ± 1.4	—
TMT (seconds)		—
A	30.6 ± 9.7	—
B	71.7 ± 36.5	—
CPZ Equivalent Doses (mg/day)	330 ± 287	—

SD: standard deviation, PANSS: positive and negative symptom scale, WTAR FS IQ: Wechsler Test of Adult Reading Full Scale Intelligence Quotient, HVLT: Hopkins Verbal Learning Test, TMT: Trail Making Test, CPZ: chlorpromazine.

### Modulations of the TEP amplitude by SICI and ICF paradigm from the DLPFC in patients with schizophrenia compared to controls

**Figure 1:**
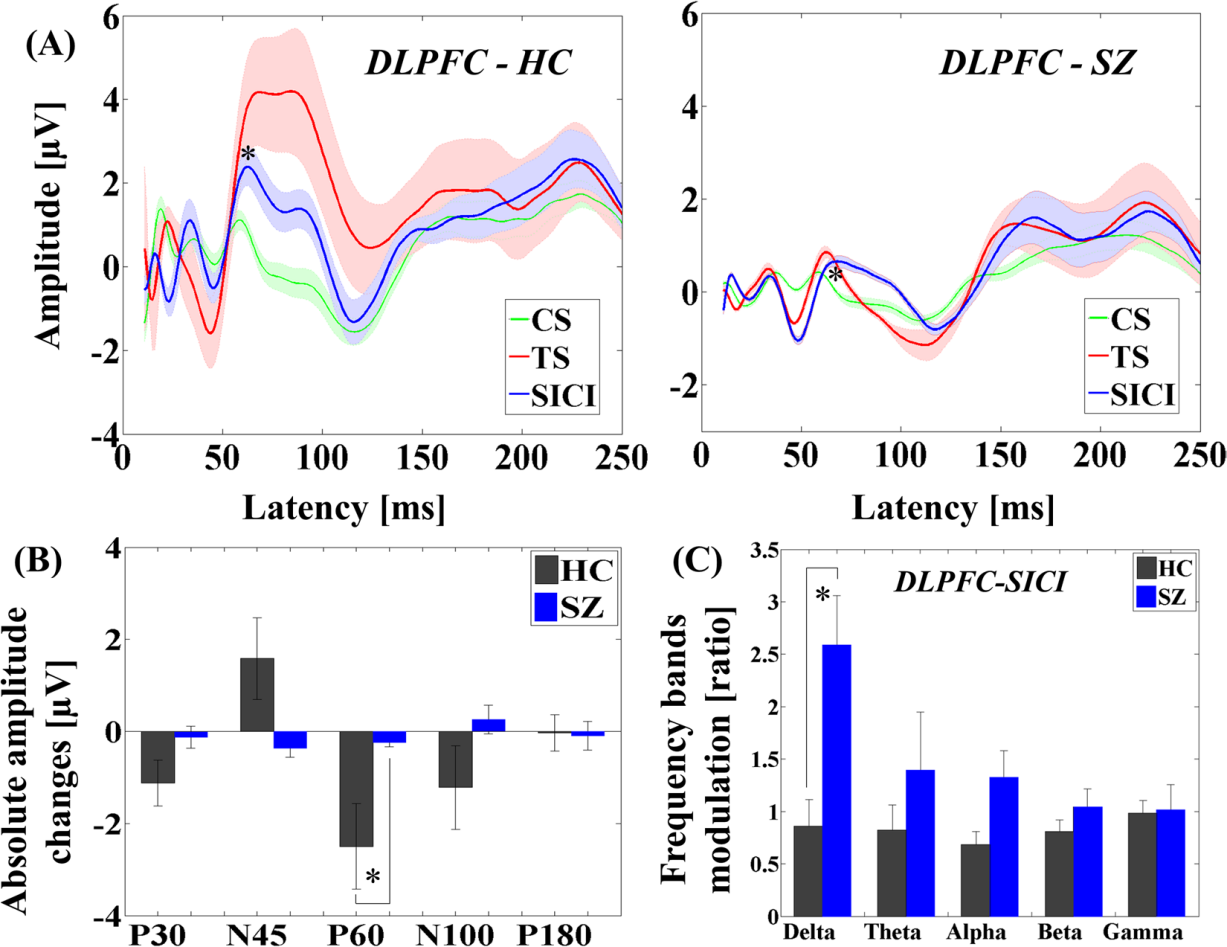
Modulation of TEPs by SICI paradigm administered TMS to DLPFC. (**A**) Group averaged TEPs at the region of interest (left DLPFC) in patients with schizophrenia following TS (red; delivered at a time equal to 0 ms), SICI (CS.TS) (blue) and CS alone (green line; delivered at −2 ms, i.e. 2 ms prior to TS) with standard error of mean (SEM) of each TEP trace. P60 TEP was significantly reduced in amplitude by SICI. (**B**) Group comparisons of TEP changes by SICI at the region of interest (left DLPFC) between healthy controls and schizophrenia patients. Patients group demonstrated significantly lower inhibitory modulation on P60 TEP compared to healthy controls. (**C**) Modulation of frequency bands by SICI in the DLPFC between healthy controls and patients with schizophrenia: patients with schizophrenia showed significantly less inhibitory modulation on delta frequency band with SICI at the region of interest (left DLPFC), compared to healthy controls. Error bars for each graph represent standard error.

The analysis of variance (ANOVA) for TEP amplitudes in the SICI paradigm showed significant main effects of diagnosis (F_1,22_ = 9.231, p = 0.006) and TEP component (F_4,88_ = 19.603, p < 0.0001) and significant TEP component × diagnosis (F_4,88_ = 3.259, p = 0.015), TMS condition × TEP component (F_4,88_ = 3.528, p = 0.010), TMS condition × TEP component × diagnosis (F_4,88_ = 4.569, p = 0.002) interactions. Post-hoc analyses for TEP amplitude changes by SICI paradigm between patients and controls demonstrated a significant difference in P60 TEP (t_22_ = −4.961, p < 0.0001), showing that SICI induced a significantly smaller reduction in the patient group. In addition, we observed a significant TEP modulation on P60 (t_11_ = 2.248, p = 0.046) by SICI in patient group (see Fig. 1(A)). Of note, we observed no main effects of group between patients and controls in SICI (F_1,22_ = 0.661, p = 0.428) as well as ICF (F_1,22_ = 0.286, p = 0.598), indicating that there were no significant differences in single-pulse TEPs between the two groups.

**Figure 2:**
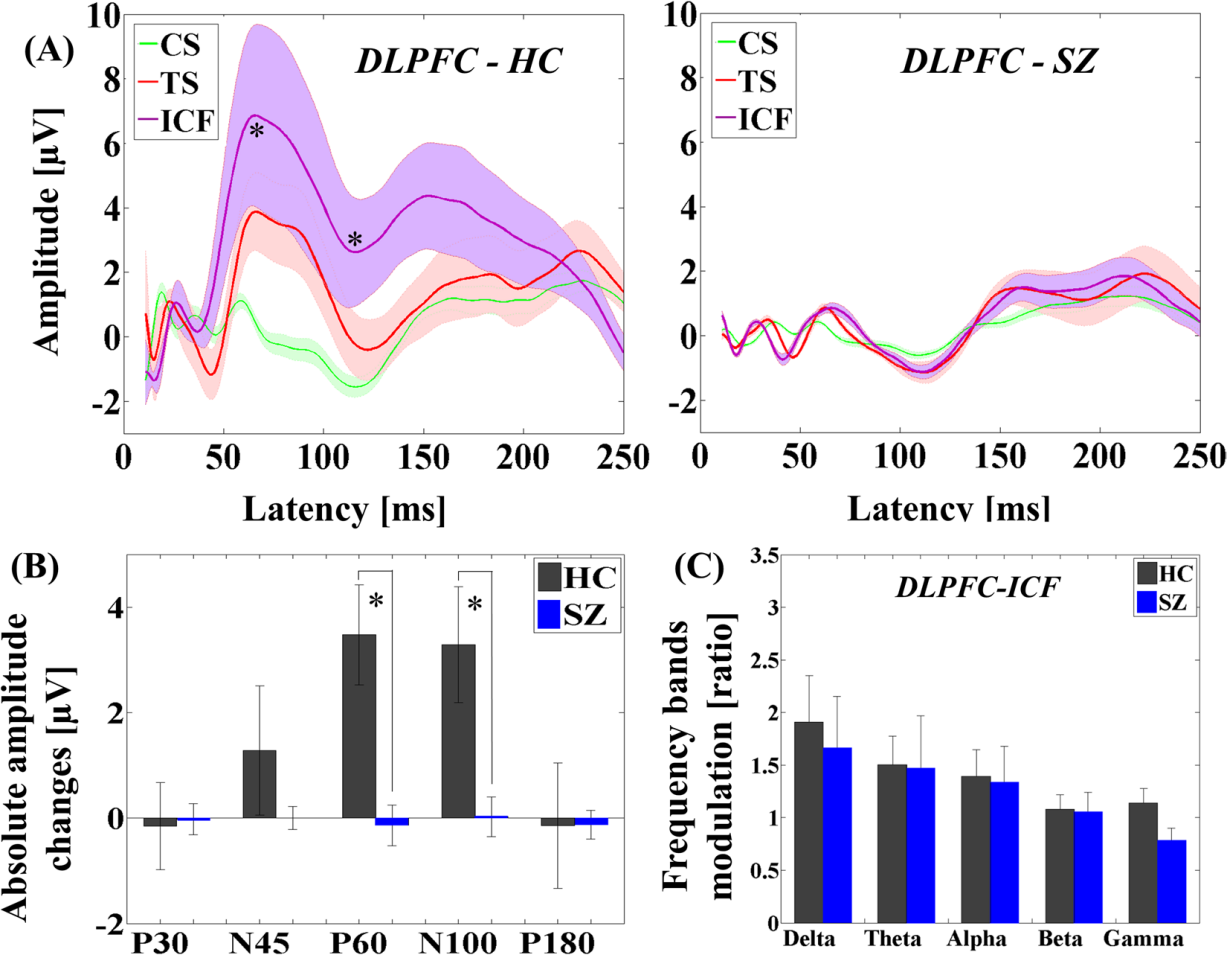
Modulation of TEPs by ICF paradigm administered TMS to DLPFC. (**A**) Group averaged TEPs at the region of interest (left DLPFC) in patients with schizophrenia following TS (red; delivered at a time equal to 0 ms) and ICF (CS.TS) (purple) and CS alone (green line; delivered at −10 ms, i.e. 10 ms prior to TS) with SEM of each TEP trace. No TEP amplitudes were significantly increased by ICF in patients group. **(B)** Group comparisons of TEP changes by ICF at the region of interest (left DLPFC) between healthy controls and schizophrenia patients. Patients group showed significantly lower facilitatory modulations on P60 and N100 TEPs compared to healthy controls. (**C**) Frequency band power modulations by ICF paradigms in the DLPFC between healthy controls and patients with schizophrenia: there was no significant difference of modulations on any frequency bands with ICF at the region of interest (left DLPFC) between the two groups. Error bars for each graph represent standard error.

On the other hand, the ANOVA for TEP amplitude in the ICF paradigm indicated significant main effects of diagnosis (F_1,22_ = 18.648, p < 0.0001), TMS condition (F_1,22_ = 12.240, p = 0.002), and TEP component (F_4,88_ = 14.991, p < 0.0001) and significant TMS condition × diagnosis (F_1,22_ = 13.494, p = 0.001), TEP component × diagnosis (F_4,88_ = 5.951, p < 0.0001), TMS condition × TEP component (F_4,88_ = 2.760, p = 0.033), and TMS condition × TEP component × diagnosis (F_4,88_ = 2.857, p = 0.028) interactions. Post-hoc analyses for TEP amplitude changes by ICF paradigm between patients and controls demonstrated significant differences in P60 (t_22_ = 5.174, p < 0.0001) and N100 (t_22_ = 3.273, p = 0.003) TEPs, showing that ICF induced significantly smaller facilitations in the patient group. Additionally, there was no significant TEP modulation by ICF in patient group itself (see Fig. 2(A)).

**Figure 3:**
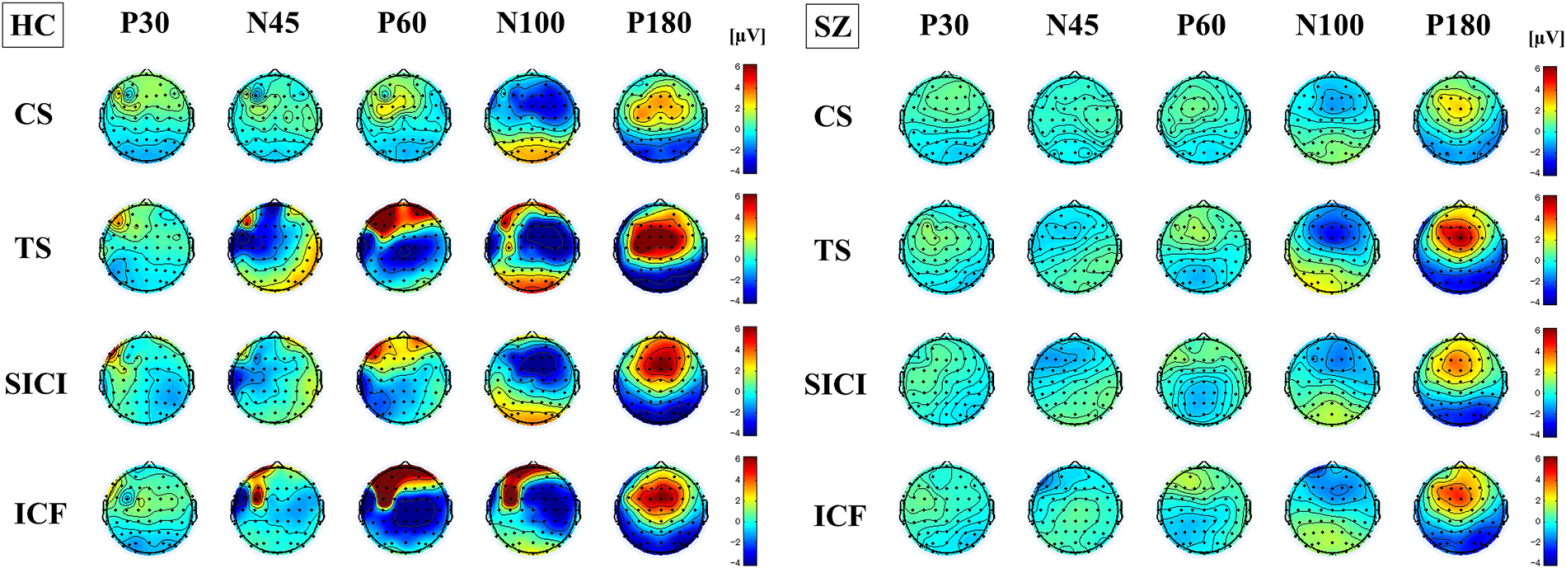
Topographical plots of paired pulse SICI and ICF paradigms. Left graph shows topographical distribution of healthy controls for each condition (CS, TS, SICI and ICF) and each TEP component (P30, N45, P60, N100, and P180). In healthy controls, TEPs by SICI reduced over the prefrontal area on P60 component, whereas TEPs by ICF increased over the left prefrontal area on P60 (i.e. more excitatory modulation) and N100 (i.e. less inhibitory modulation) components. In contrast, in right graph of patients group, the topographical changes are poor as a whole but there was a reduction of TEP N45 over the left prefrontal area.

The result of TEP traces (i.e., condition stimulus, test stimulus, and SICI condition) in patients with schizophrenia with SICI paradigm is depicted in Fig. 1(A), while the result of TEP traces in the patient group with ICF paradigm is shown in Fig. 2(A). Of note, there was no correlation between the chlorpromazine equivalent dose for each patient and the modulation of TEP components induced by SICI and ICF paradigms. Further, the results of the cross-sectional comparison analyses in SICI and ICF paradigms are shown in bar graphs of Figs 1(B) and 2(B), respectively. In addition, topographical plots depicting the TEPs for each component (i.e. P30, N45, P60, N100, and P180) and condition (CS, TS, SICI and ICF paradigms) for both groups are shown in Fig. 3. The topography of healthy controls shows the TEP reduction over the prefrontal area on P60 component (t_22_ = −4.961, p < 0.0001) in the SICI paradigm while the TEP increases over the left prefrontal area on P60 (t_22_ = 5.174, p < 0.0001) (i.e. more excitatory modulation) and N100 (t_22_ = 3.273, p = 0.003) (i.e. less inhibitory modulation) components in the ICF paradigm. In contrast, the topographical changes in patients are poor as a whole, but in the SICI paradigm, there was a reduction of TEP on N45 component over the left prefrontal area.

### Frequency band power modulations during SICI and ICF paradigms in patients with schizophrenia compared to healthy controls

**Figure 4:**
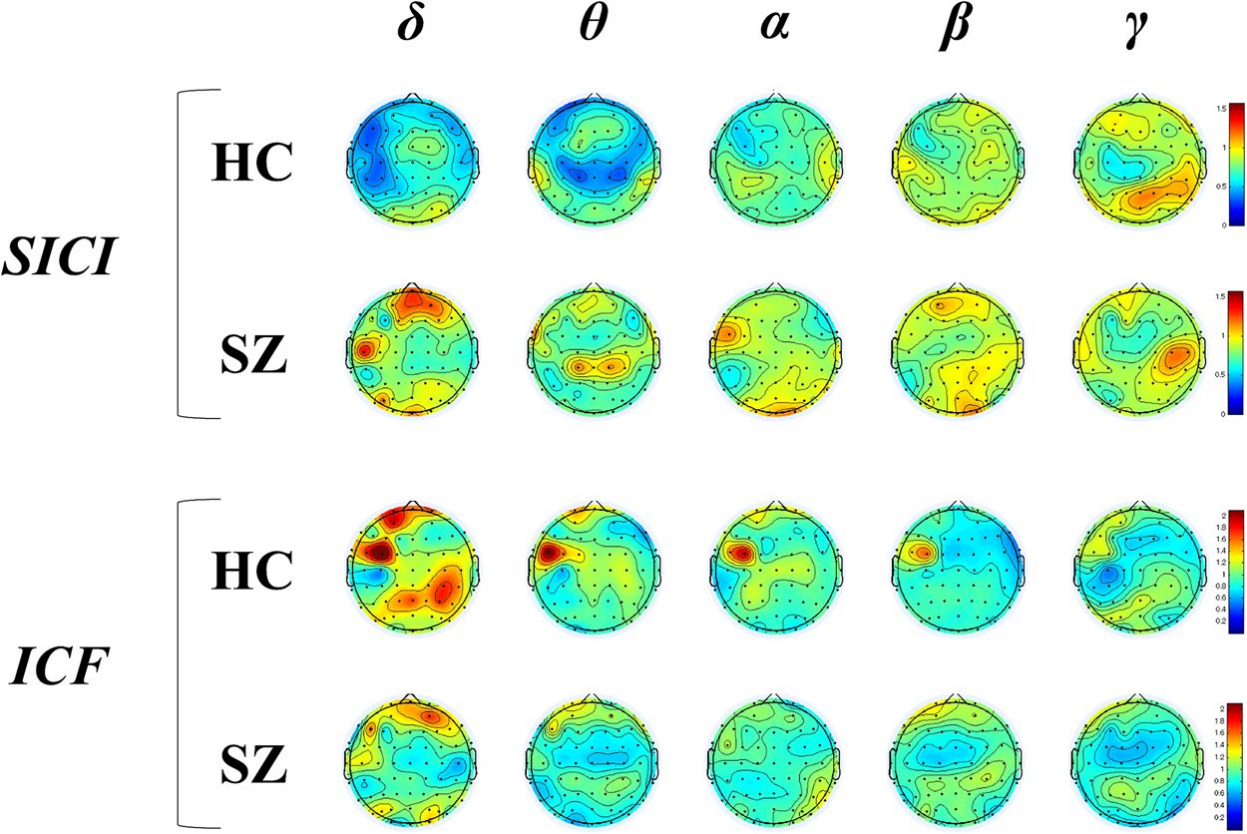
Topographical distributions of frequency band modulations by SICI and ICF paradigms. When comparing healthy controls and patients with schizophrenia, patients group showed relatively higher delta power modulation over the prefrontal area than healthy controls by SICI paradigm. In contrast, in ICF paradigm, healthy controls showed relatively higher frequency band modulations from delta to beta bands over the left DLPFC than patients with schizophrenia, but these changes were not significant.

In the SICI paradigm, there was a significant frequency band power modulation difference in the delta band (t_22_ = −3.220, p = 0.004; α = 0.05/5; controlled by the number of frequency bands) between patients and controls. By contrast, there was no significant difference in all frequency bands power modulations between the two groups with ICF (delta: t_22_ = 1.261, p = 0.221; theta: t_22_ = 0.189, p = 0.852; alpha: t_22_ = 0.446, p = 0.660; beta: t_22_ = 0.289, p = 0.775; gamma: t_22_ = 1.664, p = 0.110). These modulatory effects of SICI and ICF on frequency band powers between the healthy control group and the patient group are shown in bar graphs in Figs 1c and 2c. In addition, we plotted the topographical distributions of SICI and ICF paradigms for each frequency band modulation from delta to gamma and TEP component from P30 to P180 in both groups (see Fig. 4). Specifically, when comparing healthy controls and patients with schizophrenia, the patients showed relatively higher delta power modulation (t_22_ = −3.220, p = 0.004) over the prefrontal area compared to healthy controls in the SICI paradigm (Fig. 4 (A)). In contrast, in the ICF paradigm, healthy controls showed relatively higher frequency band modulations from delta to beta bands (δ: t_22_ = 1.261, p = 0.221; θ: t_22_ = 0.189, p = 0.852; α: t_22_ = 0.446, p = 0.660; β: t_22_ = 0.289, p = 0.775; γ: t_22_ = 1.664, p = 0.110) over the left DLPFC than patients with schizophrenia, but these changes were not significant as described above (Fig. 4(B)).

### Time-frequency analysis during SICI and ICF paradigms

**Figure 5:**
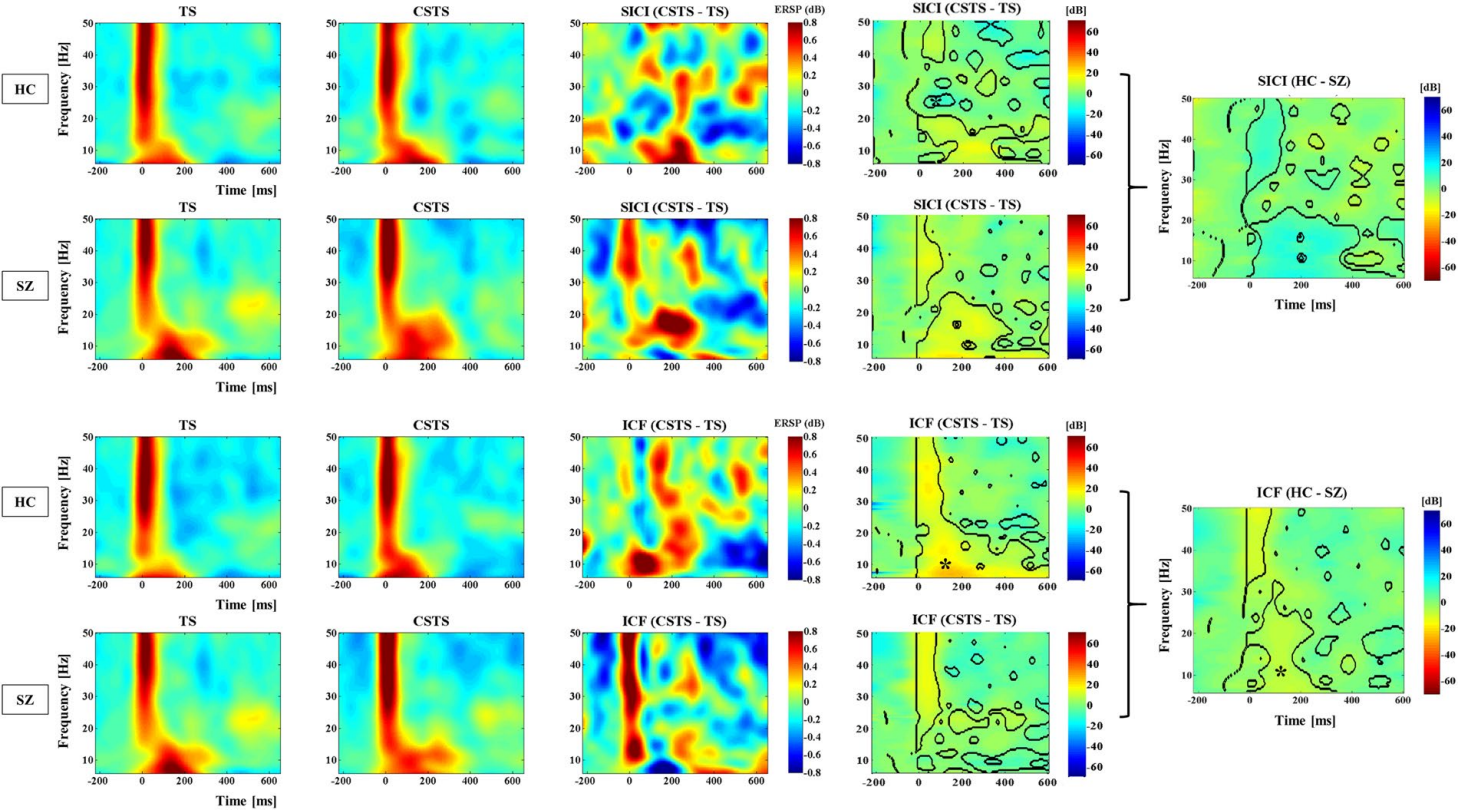
Time-frequency analysis during SICI and ICF paradigms. When comparing controls and patients, the statistical maps for the time-frequency modulation graphs during SICI as well as ICF paradigms showed significant differences in the regions inside the black dashed contours. Specifically, healthy controls showed significantly lower time-frequency modulations during SICI (blue color), compared with patients with schizophrenia, suggesting that healthy controls had a more effective suppressive effect with the SICI paradigm. In contrast, in the ICF paradigm, healthy controls showed siginificantly higher time-frequency modulations (yellow color) compared to patients, suggesting that healthy controls had a more robust excitatory response. Furthermore, the asterisks on the statistical maps in indicate the significant time-frequency modulation corresponding to the significant TEP modulations as shown in Figs 1 and 2.

When comparing controls and patients, the statistical maps for the time-frequency modulation graphs during SICI as well as ICF paradigms showed significant differences in the regions inside the black dashed contours (see Fig. 5). Specifically, healthy controls showed significantly lower time-frequency modulations during SICI (blue color), compared with patients with schizophrenia, suggesting that healthy controls had a more effective suppressive effect with the SICI paradigm. In contrast, in the ICF paradigm, healthy controls showed siginificantly higher time-frequency modulations (yellow color) compared to patients, suggesting that healthy controls had a more robust excitatory response. Furthermore, the asterisks on the statistical maps in Fig. 5 indicate the significant time-frequency modulation corresponding to the significant TEP modulations as shown in Figs 1 and 2. Thus, it is suggested that patients with schizophrenia may have less cortical inhibition in the SICI as well as less cortical excitation in the ICF in comparison with healthy controls.

### Cognitive and clinical correlations between significant TEP amplitude changes and clinical/cognitive measures in patients with schizophrenia

**Figure 6:**
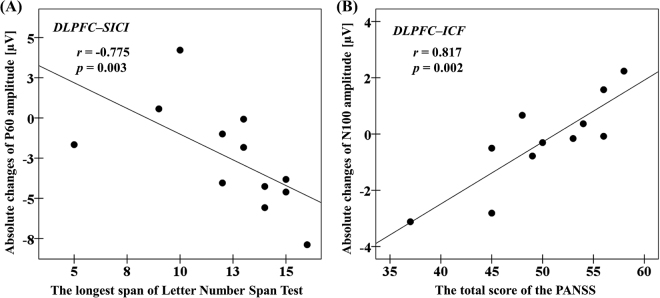
Cognitive and clinical correlations with modulation of TEPs at the left DLPFC by SICI and ICF in patients with schizophrenia. (**A**) With DLPFC-SICI paradigm, absolute changes of P60 amplitude was significantly correlated with the longest span of the Letter-Number Span Test. (**B**) With DLPFC-ICF paradigm, absolute changes of N100 amplitude was significantly correlated with the total score of the Positive and Negative Syndrome Scale (PANSS).

A highly significant correlation between TEP P60 amplitude change induced by SICI and the longest span of the Letter-Number Span Test (r = −0.775, p = 0.003, N = 12; α = 0.05/5; controlled by the number of cognitive/clinical measures) (see Fig. 6(A)) was observed in patients, while no other relationship was uncovered for measures of IQ or executive functioning. Furthermore, there was a highly significant correlation between TEP N100 amplitude change induced by ICF and the total score of the Positive and Negative Symptom Scale (PANSS) (r = 0.817, p = 0.002, N = 11; α = 0.05/5; see Fig. 6(B)).

## Discussion

The present study demonstrated modulatory effects of SICI and ICF paradigms on TEP components from the DLPFC in patients with schizophrenia compared to healthy controls. Patients with schizophrenia showed a significantly reduced inhibition of P60 TEP by SICI, suggesting that GABA_A_ receptor-mediated inhibitory function may be weakening, and significantly reduced facilitations on P60 and N100 TEPs by ICF, suggesting that glutamatergic receptor-mediated function may be weakening as well, compared to healthy controls. The inhibition of delta power with SICI was selectively impaired in patients with schizophrenia compared to healthy controls. Furthermore, in patients with schizophrenia, the degree of the modulatory effect on P60 by SICI was significantly correlated with working memory performance as assessed by longest span of the Letter-Number Span Test and also the extent of modulation on N100 by ICF was significantly correlated with the severity of the symptoms of schizophrenia as evaluated with the total score of the PANSS.

Differences in the neurophysiological modulation of TEPs induced by SICI paradigm in the DLPFC between patients and controls may add to accumulating evidence of GABA_A_ergic dysfunction in the DLPFC in schizophrenia^3,33^. Further, the modulation of P60 TEP with SICI was significantly correlated with performance of the Letter-Number Span Test, suggesting the relationship between working memory and GABA_A_ activity^34^. This finding may support at least in part of the physiological results of previous studies that have shown a relationship between GABA levels in the DLPFC and average gamma amplitude during working memory task^31^. Although the relationship between glutamatergic dysfunction and cognitive impairment is well known in schizophrenia^32^, we did not observe any significant correlation between TEP modulations by ICF and cognitive measures in the present study. A recent meta-analysis reviewing the effects of glutamate positive modulators on cognitive deficits in patients with schizophrenia demonstrated that there is no significant effect of glutamate positive modulators on cognition^35^. Thus, the cortical excitatory function as indexed by ICF, which is at least in part associate with glutamatergic receptor-mediated function, may not be directly related to cognitive dysfunction in schizophrenia. In other words, the reduced cortical excitability seen in the ICF paradigm in patients with schizophrenia may be related to the chronicity of the disease rather than the cognitive dysfunction itself.

The inhibition of delta power by SICI was impaired among patients with schizophrenia compared to healthy controls. This finding is in line with previous studies that reported increased resting EEG delta power in patients with schizophrenia compared to healthy controls^36^, which has been associated with negative symptoms of schizophrenia^37^. Taken together, our findings suggest that prefrontal delta activity may be related to neurophysiological property in terms of GABA_A_ receptor-mediated inhibitory dysfunction in patients with schizophrenia. In contrast, several studies have shown a relationship between GABA_A_ receptor-mediated inhibition and synchronized gamma oscillations *in vitro* or animal model^38,39^ as well as in human subjects^40^. Thus, initially, we anticipated that the modulation of gamma band power by the SICI paradigm in patients with schizophrenia would be lower than in healthy controls. However, our result showed no significant difference in the modulation of gamma band power by SICI between the two groups. This may be partially explained by a medication effect on gamma band power in patients with schizophrenia as antipsychotic drugs are likely to have effects on SICI, which is associated with GABA_A_ receptor-mediated inhibitory function^41^.

While some studies have reported decreased glutamate levels as measured by magnetic resonance spectroscopy in patients with chronic schizophrenia^6,42^, the evidence of glutamatergic dysfunction in schizophrenia is mixed depending on the subtype and stage of schizophrenia^43,44^. In the present study, there was no significant TEP modulations by ICF in patients with schizophrenia However, between patients and controls, there were significant differences of modulation of TEP P60 and N100 with ICF paradigm (controls > patients), suggesting that, at least in part, glutamatergic receptor-mediated function in the DLPFC may be reduced in patients with schizophrenia. Thus, these results may support previous findings^6,42^, that indicate a decrease in glutamate levels in the prefrontal cortex in patients with chronic schizophrenia.

Further, in the ICF paradigm from the DLPFC, amplitude change of the TEP N100 component was significantly correlated with the total score of PANSS in patients with schizophrenia, suggesting that patients who may have had reduced glutamatergic excitatory effect on N100 had lower clinical symptom severity. It can be speculated that patients with schizophrenia who have a lowered level of cortical excitability as indexed by ICF, may have less glutamate-mediated excitotoxicity in the prefrontal cortex^45^, that may be associated with a lower symptom burden.

As another important factor contributing to schizophrenia, it is well known that dopaminergic modulation plays a significant role in cognitive functions of the prefrontal cortex including functions that are impaired in patients with schizophrenia^46^. Specifically, on a cellular level, dopamine modulates pyramidal cell excitability through its actions on local circuits of GABAergic interneurons in the prefrontal cortex^47–49^. Dopamine suppresses glutamatergic excitatory transmission between pyramidal neurons through activation of D1 receptors that induce an increase in first spiking interneuron excitability, which in turn results in enhanced GABAergic inhibitory transmission to pyramidal neurons in the prefrontal cortex^49^. Therefore, dysfunction of tuning due to changes in dopamine and/or GABA transmission might also underlie deficits in cognitive impairments in schizophrenia^3,46,50^.

Furthermore, in connection with the excitatory function by ICF and the inhibitory function by SICI, previous studies have shown that there is a balanced E/I relationship between GABA receptor-mediated inhibition and glutamate receptor-mediated excitation in the healthy brain and this property is thought to be crucial for information processing^20,51^. However, it has been suggested that patients with schizophrenia may have impaired E/I balance in neural circuits in the prefrontal cortex^52^, leading to deficits in cognition and social behavior^20,53,54^. The present finding may support the characteristics of E/I balance in the prefrontal cortex between healthy controls and patients with schizophrenia. Moreover, in the patients group, amplitude changes of P60 component by SICI was correlated with performance of working memory, as well as amplitude changes of N100 component by ICF was correlated with severity of clinical symptoms. Therefore, taken together these clinical and cognitive correlations with the neurophysiological measures of inhibitory effect on P60 and excitatory effect on N100, as measured with SICI and ICF paradigms, respectively, may represent a pathophysiological mechanism underlying the symptoms of patients with schizophrenia.

Some limitations exist in the present study. First, concomitant medications (Supplementary Table) may have potential confounding effects on SICI and ICF in the patient group. However, no participant took benzodiazepines or glutamate modulators in the present study. In terms of potential confounding of SICI and ICF by dopamine antagonists, although a previous study has reported that haloperidol has negative impact on SICI and ICF^55^, there is no evidence that other dopamine antagonists have direct effects on SICI and ICF. In the present study, none of the patients were taking haloperidol. Furthermore, there was no relationship found between chlorpromazine equivalent doses and the extent of inhibition or excitation suggesting that the present results cannot readily be explained by the use of antipsychotic medication. Nonetheless, it is important to determine the effect of medication on these measures^56^. A second limitation to this study is that cognitive assessments were only administered in the patient group and not in the controls. The relationship between deficits in cognition and neurotransmission was of greater interest in the patient group in the present study. It was anticipated that ceiling effects in healthy controls would have limited the power to detect such relationships. Thirdly, since we did not conduct a pharmaco-TMS-EEG study, there is a limit that it cannot show direct evidence from our results. Lastly, we did not use an auditory masking noise during the experiments. Thus, there may be some potential effect of auditory input associated with click sounds of the TMS coil. However, at the stage of off-line analysis, we compared test TMS pulse and conditioned TMS pulse (i.e. SICI or ICF) that had nearly identical auditory inputs associated with the click sounds. As a result, this potential confounding may be minimal. Nonetheless, future studies may examine the relationship between SICI and ICF and cognition and whether this effect is selective to the working memory domain as we observed in the patient group.

In conclusion, the present study adds to the evidence for GABA_A_ receptor-mediated inhibitory as well as glutamate receptor-mediated excitatory dysfunctions in schizophrenia and suggests possible pathophysiological mechanism in the emergence of clinical and cognitive symptom of the illness. Using combined TMS-EEG, it is possible to further investigate GABA receptor-mediated inhibitory and glutamate receptor-mediated excitatory neurotransmissions as possible targets or mechanisms through which treatments may exact their therapeutic effect may help to advance the treatment of this devastating disorder. The present study demonstrates the applicability of the SICI-ICF paradigms using combined TMS–EEG. Moreover, the E/I balance and its role in cognition may serve as a possible dimension to investigate across mental illness within the Research Domain Criteria (RDoC) framework^57^.

## Materials and Methods

### Participants

Twelve right-handed patients with schizophrenia (8 males, mean age: 41 ± 10 years) and 12 healthy control participants (6 males, mean age: 39 ± 12 years) were examined in the present study. SICI and ICF paradigms were administered to the DLPFC using a combined TMS–EEG in all participants. Participants were eligible for this study if they met the following criteria: (i) between ages 18 and 59; (ii) no history of neurological disorders including seizure, syncope or stroke; (iii) no current (past 6 months) of alcohol or other drug abuse or dependence; (iv) current abstinent non-smoker verified by a carbon monoxide reading (less than or equal to 4 ppm of a carbon monoxide (CO) level as measured by the breath CO monitor)^58^. For the healthy controls, they also met the following criteria: v) no history of neuropsychiatric disorders; or vi) no prescription medications. For patients with schizophrenia, they met the criteria as follows: vii) clinically stable determined by the PANSS score of ≤70; (viii) no anticholinergic drugs, benzodiazepines, or glutamate modulators; (ix) had not been hospitalized in the past 3 months, and were on a stable dose of antipsychotic medications for at least one month. CPZ equivalents were calculated according to established methods^59^. All participants were screened with the Structured Clinical Interview for DSM–IV Axis I Disorders prior to study participation. Written informed consent was obtained from each participant. The experiment was conducted in accordance with the Declaration of Helsinki and was approved by the Research Ethics Board of the Centre for Addiction and Mental Health.

### Clinical and cognitive assessment for patients with schizophrenia

To assess the severity of clinical symptoms, 11 out of 12 patients were examined with the PANSS (1 patient could not complete the PANSS). For cognitive measures, the Wechsler Test of Adult Reading (WTAR), the Letter-Number Span Test, and the Trail Making Test (TMT) Parts A & B, the Hopkins Verbal Learning Test (HVLT) were performed for patients with schizophrenia.

### TMS procedure and EMG measure

Monophasic TMS pulses were administered to the left primary motor cortex using a 70 mm figure–of–eight coil, and two Magstim 200 stimulators (Magstim Company Ltd., UK) connected via a Bistim module. During the TMS testing, participants sat in a chair with their eyes open and right hand relaxed throughout the study. First, the primary motor cortex hot spot for the right first dorsal interosseous muscle to evoke the largest motor evoked potential (MEP) was determined. Second, the individual intensity of resting motor threshold (RMT) to induce a minimum of 50 μV MEP amplitude from the right first dorsal interosseous muscle 5 times out of 10 trials was determined. Subsequently, the individual intensity to induce 1 mV peak–to–peak MEP amplitude of the same muscle was determined. The RMT and the intensity to induce 1 mV peak-to-peak MEP amplitude were assessed to determine the intensity parameters of SICI and ICF paradigms.

### SICI and ICF measures

SICI and ICF were examined according to established methods^8^; specifically, the inter-stimulus interval (ISI) of SICI was set to 2 ms while the ISI of ICF was set to 10 ms. Conditioning stimulus (CS) intensity was set at 80% of RMT and test stimulus (TS) intensity was set at the intensity to evoke a 1 mV peak-to-peak MEP amplitude when TMS was delivered alone. The left DLPFC target was individually determined based on the EEG cap navigated F5 electrode site method^60^.

### EEG recording and pre–processing

EEG was acquired through a 64–channel Neuroscan Synamps 2 EEG system with TMS-compatible EEG cap (Compumedics Neuroscan, Australia). All electrodes were referenced to an electrode placed on the vertex. Recording electrodes impedance was kept lowered to ≤5 kΩ during experiment. EEG signals were recorded at DC with a sampling rate of 20 kHz and an online lowpass filter of 200 Hz was applied. EEG data were processed offline using the MATLAB software (R2014a, The MathWorks, MA, USA). All data were down–sampled to 1000 Hz for analyses.

### EEG signal processing

EEG signal processing was performed in line with published methodology^30^. The continuous EEG data were epoched from −1000 ms to 2000 ms relative to the TMS pulse. Baseline correction was conducted with respect to the pre-stimulus interval −500 ms to −110 ms. To avoid TMS artifacts, the epoched EEG data was re-segmented from 10 ms to 2000 ms post-TMS. Then, EEG data were visually inspected to exclude trials and channels that were highly contaminated with noise. As a result, more than 80% of trials and 95% of channels survived artifact rejection. Subsequently, independent component analysis (ICA) was applied to minimize and remove the typical TMS–related decay artifacts as well as eye–related and remaining muscle activity related components. Following the ICA cleaning, the Butterworth, zero-phase shift 1–55 Hz band pass filter (24 dB/Oct) and notch filter were applied. In each subject, the number of ICA components that were removed from original 62 ICA components was no greater than 20%. Finally, data was re-referenced to the average for further analyses.

### TMS-evoked potential analyses of SICI and ICF paradigms

For the EEG analyses of SICI and ICF, TEP induced by each paradigm was analyzed individually. Specifically, the influence of each paradigm on the individual TEP components (P30, N45, P60, N100 and P180) value was computed for each condition (CS, TS, and CS.TS) at the left DLPFC region obtained from the SICI and ICF experiments from the DLPFC. We extracted the maximum/minimum amplitude values for each positive/negative deflections individually for each electrode included in the left DLPFC region. Then, values were averaged. In addition, we calculated the TEP traces over a time window of 10–250 ms after TMS pulse, but specific time windows of each TEP component were not set for the analyses because we extracted the amplitude peak and trough values manually by visual inspection for each subject. Further, we clustered the specific electrodes as regions of interest for analyses of the left DLPFC (Fp1, AF3, AF7, F1, F3, F5, F7, FC1, FC3, FC7) regions according to our previously published method^30^, in order to ensure consistency with previous analytical method in healthy controls and to reduce the influence of contaminated electrode channel data under the TMS coil. We created the TEP traces of each SICI and ICF paradigm for both healthy controls and patients with schizophrenia, bar graphs comparing the TEP results for both groups, and topographical plots of each paired pulse TEPs for each TEP component in both groups.

### Frequency band powers analyses during SICI and ICF paradigms

Frequency band power changes induced by SICI and ICF paradigms were calculated by applying the Hilbert transform method to TEP focused on the left DLPFC and each frequency band was defined as follows: delta (1–3 Hz); theta (4–7 Hz); alpha (8–14 Hz); beta (14–30 Hz); and gamma (30–50 Hz). Then, modulations of each frequency band were calculated as a ratio of frequency band power of CS.TS over TS condition (i.e., ratio = CS.TS/TS) for both SICI and ICF paradigms in healthy controls and patients with schizophrenia groups. We created bar graphs comparing the frequency band power modulation of TEPs and topographical plots for each frequency band (i.e., delta, theta, alpha, beta, and gamma bands) and each TEP component (i.e., P30, N45, P60, N100, and P180) during the SICI and ICF paradigms in both groups.

### Time-frequency analysis for SICI and ICF paradigms

Time-frequency analysis for SICI and ICF paradigms from the left DLPFC for both groups was performed to evaluate the difference of time-frequency patters qualitatively between the two groups. Specifically, the wavelet frequency analysis was applied via “Event related spectral perturbation” (ERSP) using the newtimef() function in the open source toolbox EEGLAB on the average of each trial for each condition for all subjects. The wavelet cycle was set to [30.5] and a period from 400 ms before and 1000 ms after the TMS pulse was analyzed. The baseline activity was subtracted and the ERSP was expressed in dB as 10*log10(R) where R is the ratio of power between the signal and its baseline period.

### Statistical analyses

IBM SPSS Statistics 19 (Armonk, New York, USA) was used for statistical analysis. The following assessments were performed: 1) SICI or ICF effects on TEP amplitudes between patients with schizophrenia and healthy controls; 2) correlation analyses in patients with schizophrenia: i) between amplitude changes of TEP components induced with SICI and ICF and clinical and cognitive measures; and ii) between significant amplitude changes of TEPs by SICI and ICF; and 3) modulatory effects by SICI and ICF on frequency bands between patients and controls. ANOVAs were applied to TEP amplitudes to examine the significant effects of SICI and ICF on TEPs, separately. Specifically, we performed a three–way ANOVA with TEP component (P30, N45, P60, N100, and P180) and TMS condition (test pulse and SICI or ICF) as within-subject factors and diagnosis (patients vs controls) as a between-subject factor for each SICI and ICF paradigm. Cross-sectional comparison analyses were performed for TEP amplitude changes by SICI or ICF by using post–hoc independent t–tests. We performed correlation analyses with Pearson’s correlation coefficient for the significant results obtained from above ANOVA. Pearson’s correlation was applied based on the assumption of normal distribution calculated by the Shapiro-Wilk test. A significant level of α = 0.05 was applied. Further, modulatory effects on the frequency band powers from delta to gamma over the left DLPFC during the SICI and ICF paradigms were exploratory examined with independent t–tests compared to the healthy control group with Bonferroni correction (α = 0.05/5). In addition, with respect to the time-frequency analysis for SICI and ICF paradigms over the left DLPFC, we applied the EEGLAB bootstrapping statistics (N = 1000) with an alpha value set at 0.01. Further the false discovery rate (FDR)-controlling procedures was used to correct multiple comparisons.

## Electronic supplementary material

Supplementary table
