# Supplementary material for: Evaluation of short interval cortical inhibition and intracortical facilitation from the dorsolateral prefrontal cortex in patients with schizophrenia
Source: Sci Rep. 2017 Dec 6;7:17106. doi: 10.1038/s41598-017-17052-3 (PMC5719013; doi:10.1038/s41598-017-17052-3)
Supplement: Supplementary file 1 — Supplementary table [file 41598_2017_17052_MOESM1_ESM.pdf]

Evaluation of short interval cortical inhibition and intracortical facilitation from the dorsolateral prefrontal cortex in patients with schizophrenia

Yoshihiro Noda[1,2], Mera S. Barr[1,2,3], Reza Zomorrodi[1], Robin F. H. Cash[4], Faranak Farzan[1,2,3], Tarek K. Rajji [1,2,3], Robert Chen[4], Zafiris J. Daskalakis[1,2,3], Daniel M. Blumberger[1,2,3*]

[1]Temerty Centre for Therapeutic Brain Intervention, Centre for Addiction and Mental Health, Toronto, Ontario, M6J 1H4, Canada

[2]Department of Psychiatry, University of Toronto, Toronto, Ontario, M5T 1R8, Canada

[3]Campbell Family Mental Health Research Institute, Centre for Addiction and Mental Health, Toronto, Ontario, M5T 1R8, Canada

[4]Division of Neurology, Department of Medicine, University of Toronto, Division of Brain, Imaging and Behaviour – Systems Neuroscience, Krembil Research Institute, University Health Network, Toronto, M5T 2S8, Ontario, Canada

*Corresponding author

Address Correspondence to:

Daniel M. Blumberger, MD, MSc, FRCPC

Medical Head and Co-Director, Temerty Centre for Therapeutic Brain Intervention

Head, Late-Life Mood Disorders Clinic

Clinician Scientist, Campbell Family Mental Health Research Institute

Centre for Addiction and Mental Health

Associate Professor, Department of Psychiatry

University of Toronto

1001 Queen St. W. Unit 4-115

Toronto, ON, M6J 1H4

Email: daniel.blumberger@camh.ca

Phone 416-535-8501 x33662

Supplementary table. The breakdown of psychotropic medications of the patients

Antipsychotics	Antidepressants	Mood stabilizers
clozapine (4)	***citalopram (2)***	***lamotrigine (1)***
olanzapine (3)	***sertraline (1)***	
risperidone (2)	***venlafaxine (1)***	
paliperidone (1)	***escitalopram (1)***	
lurasidone (1)		
aripiprazole (1)		
quetiapine (1)		

The number in parentheses indicates the number of patients who had been administered that medication.
